# Pre-stroke glycemic variability estimated by glycated albumin predicts hematoma expansion and poor outcomes in patients with spontaneous intracerebral hemorrhage

**DOI:** 10.1038/s41598-023-40109-5

**Published:** 2023-08-08

**Authors:** Sang-Hwa Lee, Jong-Hee Sohn, Chulho Kim, Yeo Jin Kim, Jin Pyeong Jeon, Jinseo Yang, So Young Park, Hyuk Jai Choi

**Affiliations:** 1 Department of Neurology, Hallym University Chuncheon Sacred Heart Hospital, Chuncheon, South Korea; 2 Department of Neurology, Kangdong Sacred Heart Hospital, Seoul, South Korea; 3 Department of Neurosurgery, Hallym University Chuncheon Sacred Heart Hospital, Chuncheon, South Korea; 4 Department of Endocrinology and Metabolism, Kyung Hee University Hospital, Seoul, South Korea; 5 Institute of New Frontier Research Team, Hallym University, Chuncheon, South Korea

**Keywords:** Neurology, Risk factors

## Abstract

Glycemic variability has been shown to be correlated more with oxidative stress than chronic hyperglycemia. We evaluated the impact of pre-stroke glycemic variability measured using glycated albumin (GA) on hematoma expansion and clinical outcomes following spontaneous intracerebral hemorrhage (ICH). We consecutively enrolled 343 patients with ICH for 72 months using a single-center registry database. The primary outcome measure was hematoma expansion. The secondary outcome measures were early neurological deterioration (END), 1-month mortality, and 3-month poor functional outcomes (modified Rankin scale score of 4–6). The patients were divided into two groups based on pre-stroke glycemic variability: a higher GA group (GA ≥ 16.0%) and a lower GA group (GA < 16.0%). During the study period, there were 63 (18.4%) events of hematoma expansion, 61 (17.8%) of END, 45 (13.1%) of 1-month mortality, and 45 (13.1%) of 3-month poor functional outcomes after ICH. The higher GA group (36.4%) had higher rates of hematoma expansion, END, 1-month mortality, and 3-month poor functional outcomes than the lower GA group. Multivariate analysis showed that a higher GA level was significantly associated with increased hematoma expansion (adjusted odds ratio 5.83; 95% confidence interval [CI] 2.58–13.19, p < 0.001). The area under the receiver operating characteristic curve of GA (0.83; 95% CI 0.48–0.65) for predicting hematoma expansion was higher than that of glycated hemoglobin (0.57; 95% CI 0.48–0.65, p for DeLong’s pairwise comparison < 0.001). Higher GA levels could be a reliable marker for predicting hematoma expansion and poor outcomes following ICH.

## Introduction

Hematoma expansion can occur in approximately 26% of patients with intracerebral hemorrhage (ICH) within 1 h of hospitalization, with a further 12% of patients with ICH developing hematoma expansion within 24 h^1^. As hematoma expansion can lead to mortality and poor functional outcomes after ICH, early prevention and prediction of hematoma expansion are necessary to provide clinical benefits that would improve clinical outcomes following ICH^2–4^.

Hyperglycemia after ICH is well evaluated as a strong potential marker of hematoma expansion and outcomes. Hence, several studies have shown that glycated hemoglobin (HbA1c) and post-stroke hyperglycemia could be associated with worsening clinical outcomes after ICH by several pathomechanisms^5–7^. However, previous experimental and clinical studies have suggested that glycemic variability may induce worse effects through oxidative stress and inflammatory reactions than those produced by chronic hyperglycemia^8,9^. Therefore, we speculated that rapid detection of glycemic variability in ICH cases at the acute stage could help to establish a prevention strategy for hematoma expansion.

There is still little consensus on the gold standard method to measure glycemic variability in clinical practice and research. Traditional methods to measure glycemic variability include estimating standard deviation (SD) and coefficient of variation (CV) of the mean blood glucose level, and continuous glucose monitoring^10,11^. However, these methods require glucose data from at least two consecutive days after hospitalization for estimation; therefore, they may not provide suitable indicators for pre-stroke glycemic variability to predict hematoma expansion in emergent practice.

Glycated albumin (GA) reflects glycemic variability within four weeks; therefore, it is already widely used to monitor the glycemic status in patients with diabetes^12,13^. As GA is not influenced by various medical conditions (such as hematologic disorder, and chronic renal disease) that affect a large number of patients with stroke, GA could be a reliable marker for monitoring pre-stroke glycemic variability^14^. Moreover, considering association between GA and HbA1c, the ratio of GA to HbA1c (GA/HbA1c ratio) could reflect more accurate glycemic control^15^. Therefore, we assumed that GA may be a reliable marker for predicting glycemic variability prior to ICH. In addition, GA can be measured quickly and easily with one blood sample in an emergent setting. Similarly, several recent studies consistently showed the usefulness of GA as a marker of stroke outcomes in various cases^16–19^.

We hypothesized that high glycemic variability prior to ICH, defined by higher GA levels, could increase the risk of hematoma expansion and poor functional outcomes after ICH. Using a consecutive single-center registry database, we aimed to evaluate the impact of GA, which reflects pre-stroke glycemic variability, on hematoma expansion and clinical outcomes after ICH.

## Results

**Figure 1:**
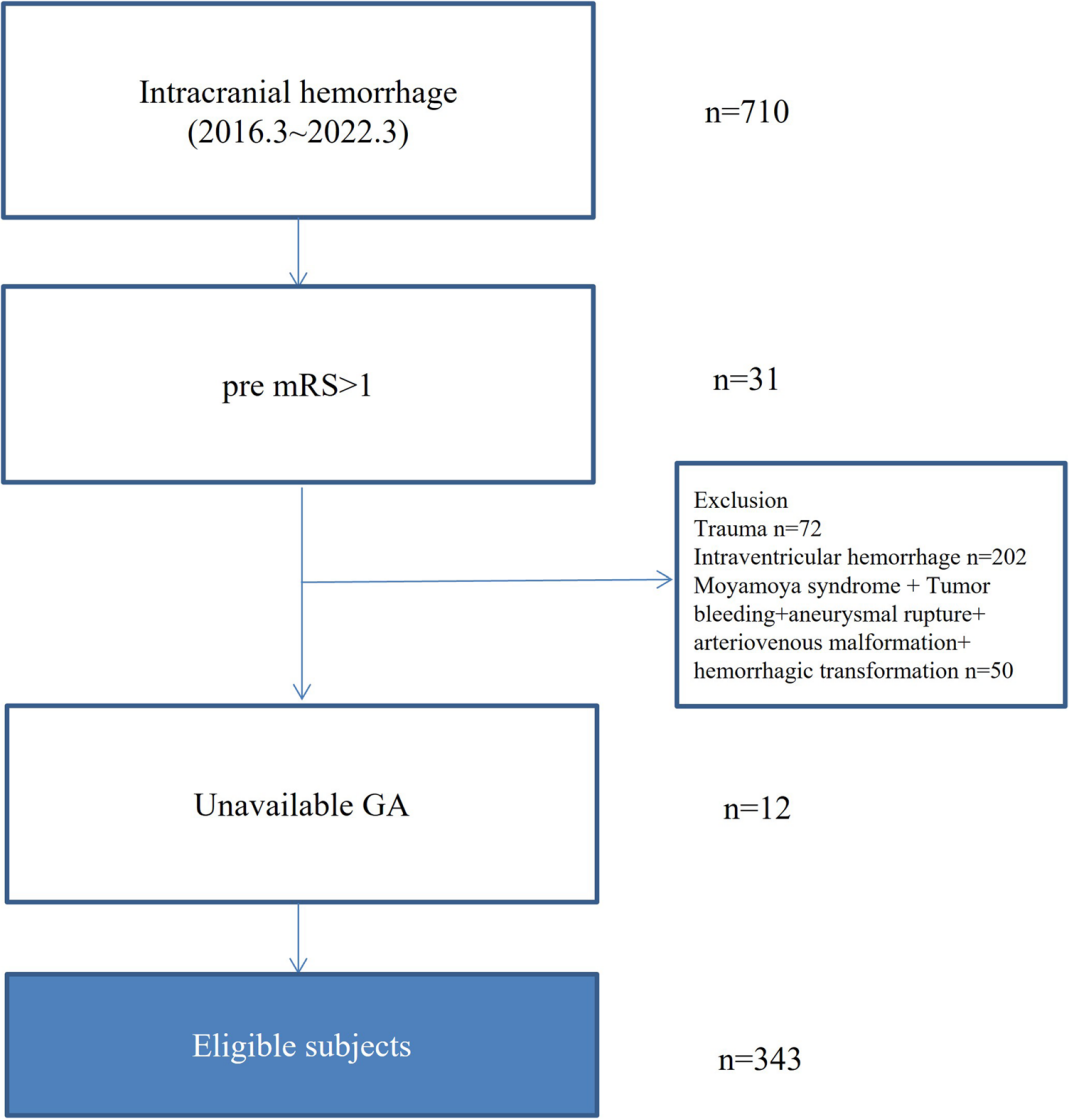
Flow chart of study. *mRS* modified Rankin scale, *GA* glycated albumin.

Among the 711 consecutively registered patients with intracranial hemorrhage, 343 patients with spontaneous ICH were enrolled in this study (mean age 64.3 ± 15.4, male 60.6%). The mean HbA1c level was 6.0 ± 1.0%, and the mean GA level was 16.8 ± 5.3% (Fig. 1). The overall rate of hematoma expansion was 18.4% (n = 63). Regarding secondary outcome measures, END was observed in 61 patients (17.8%), 1-month mortality in 45 (13.1%), and poor functional outcomes in 171 patients (49.9%). Of the 343 patients, 125 (36.4%) were in the higher GA group (GA ≥ 16%). The higher GA group was more likely to be older and have a history of stroke and diabetes mellitus (DM), higher initial random glucose levels, and higher HbA1c levels than the lower GA group. The other laboratory findings (including hs-CRP, creatinine, hemoglobin and platelet count) were not different between two groups. In addition, higher GA group had lower initial GCS score and higher NIHSS score than lower GA group (Table 1).

**Table 1:** Baseline characteristics according to GA level.

	GA < 16.0% (n = 218)	GA ≥ 16.0% (n = 125)	P-value
Age, year (SD)	61.3 (16.3)	69.5 (12.0)	0.002^†^
Male, n (%)	136 (62.4)	72 (57.6)	0.42*
Prior stroke, n (%)	26 (11.9)	38 (30.4)	< 0.001*
Hypertension, n (%)	111 (50.9)	76 (60.8)	0.09*
Diabetes mellitus, n (%)	20 (9.2)	63 (50.4)	< 0.001*
Hyperlipidemia, n (%)	16 (7.3)	15 (12.0)	0.17*
CAD, n (%)	12 (5.5)	13 (10.4)	0.13*
Current smoking, n (%)	28 (12.8)	13 (10.5)	0.61*
Prior antithrombotics, n (%)	53 (24.3)	38 (30.4)	0.25*
Initial GCS, score (IQR)	13 (7–15)	10 (6–14)	0.02^‡^
Initial NIHSS, score (IQR)	5 (4–14)	8 (5–15)	< 0.001^‡^
Lesions, n (%)			0.22*
Deep	138 (63.3)	67 (53.6)	
Lobar	47 (21.6)	34 (27.2)	
Infratentorial	24 (11.0)	14 (11.2)	
IVH	9 (4.1)	10 (8.0)	
Initial hematoma volume, mL (IQR)	12 (3–40)	10 (5–50)	0.70^‡^
Follow-up hematoma volume, mL (IQR)	12.0 (3.0–31.0)	15.0 (5.0–50.0)	0.049^‡^
Laboratory test			
Hemoglobin, mg/dL (SD)	13.7 (2.1)	13.0 (2.0)	0.97^†^
Platelet count, uL/10^3^ (SD)	222.0 (70.0)	208.0 (82.7)	0.18^†^
INR, (IQR)	1.05 (1.00–1.11)	1.06 (1.01–1.13)	0.15^‡^
LDL, mg/dL (SD)	88.8 (36.1)	78.6 (30.9)	0.10^†^
Initial glucose, mg/dL (SD)	138.2 (45.4)	195.7 (84.8)	< 0.001^†^
Creatinine, mg/dL (SD)	1.2 (3.0)	1.3 (1.8)	0.50^†^
hs-CRP, mg/dL (SD)	11.2 (26.7)	13.57 (31.5)	0.31^†^
HbA1c, % (SD)	5.6 (0.5)	6.8 (1.3)	< 0.001^†^
SBP, mmHg (SD)	160.3 (34.4)	161.2 (34.8)	0.74^†^
DBP, mmHg (SD)	79.57 (18.38)	79.76 (19.43)	0.08^†^
SBP at f/u CT, mmHg (SD)	113.9 (17.0)	114.9 (18.1)	0.24^†^

*GA* glycated albumin, *CAD* coronary artery disease, *GCS* Glasgow coma scale, *NIHSS* National Institute of Health Stroke Scale, *IVH* intraventricular hemorrhage, *INR* international normalized ratio, *LDL* low density lipoprotein, *hs*-*CRP* high sensitive C-reactive protein, *HbA1c* glycated hemoglobin, *SBP* systolic blood pressure, *DBP* diastolic blood pressure, *f/u* follow-up, *CT* computed tomography.

*Calculated using the chi-square test.

^†^Calculated using Student’s *t* test.

^‡^Calculated using the Mann–Whitney *U* test.

**Figure 2:**
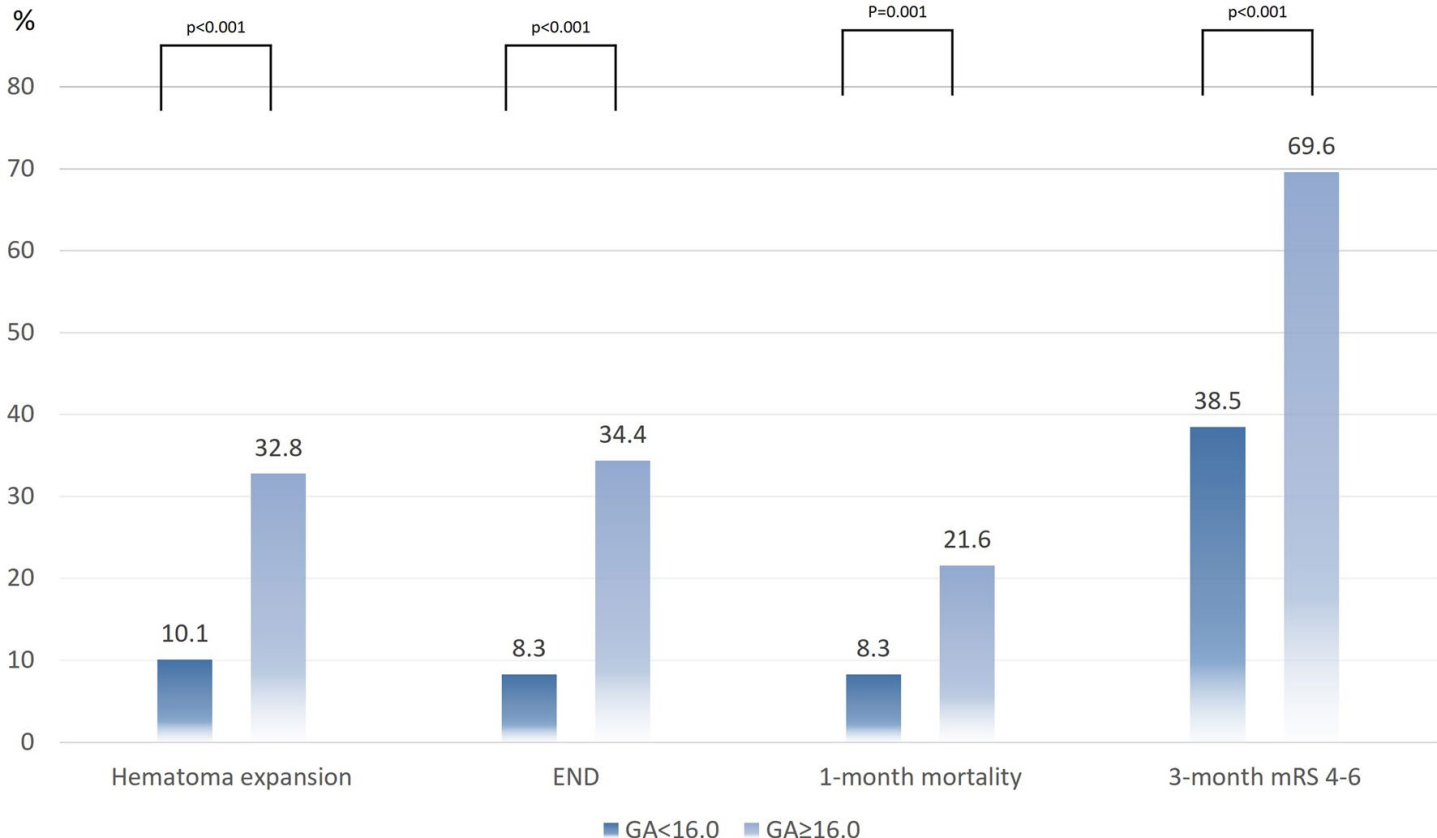
Distributions of outcomes according to GA level. *GA* glycated albumin, *END* early neurological deterioration, *mRS* modified Rankin scale.

The proportion of hematoma expansion was greater in the higher GA group than in the lower GA group (10.1% vs. 32.8%, p < 0.001). In addition, the proportions of END, 1-month mortality, and 3-month poor functional outcomes were significantly higher in the higher GA group (Fig. 2).

Multivariate analysis showed that a higher GA level (GA ≥ 16.0%) was associated with an increased risk of hematoma expansion (adjusted OR 95% CI 5.83 [2.58–13.19], p < 0.001). Concerning secondary outcome measures, a higher GA level (GA ≥ 16.0%) was associated with an increased risk of END, 1-month mortality, and 3-month poor functional outcomes (Table 2). As a sensitivity analysis, the raw GA level and GA/HbA1c ratio were also associated with increased hematoma expansion and other secondary outcomes (Table 3, Supplementary Tables 1 and 2). Moreover, HbA1c ≥ 6.5% was associated with hematoma expansion and END but not with 1-month mortality and 3-month poor functional outcomes (Table 3 and Supplementary Table 3).

**Table 2:** Multivariate analysis showing impact of higher GA on hematoma expansion and other stroke outcomes.

	Hematoma expansion		END		1-month mortality		3-month mRS 4–6	
	OR	95% CI	OR	95% CI	OR	95% CI	OR	95% CI
GA ≥ 16.0%	5.83	2.58–13.19	3.30	1.41–7.76	5.15	1.52–17.45	4.03	1.35–12.06
Age	0.99	0.96–1.01	1.01	0.98–1.03	1.05	1.01–1.10	1.04	1.1–1.08
Male	0.91	0.46–1.81	0.72	0.35–1.47	2.28	0.72–7.18	1.72	0.73–4.06
Initial GCS	0.82	0.61–1.09	0.80	0.59–1.09	0.50	0.30–0.82	0.47	0.30–0.72
Initial NIHSS	0.89	0.73–1.09	0.84	0.68–1.04	0.97	0.76–1.25	0.96	0.70–1.32
Prior stroke	0.39	0.16–0.95	0.52	0.21–1.29	0.65	0.19–2.26	0.96	0.31–3.04
Hypertension	1.89	0.93–3.81	1.82	0.87–3.82	0.93	0.29–2.94	0.44	0.19–1.07
Diabetes mellitus	0.93	0.41–2.09	1.43	0.65–3.16	1.16	0.32–4.19	2.94	1.01–8.51
Prior antithrombotics	1.39	0.65–3.00	0.59	0.26–1.34	2.25	0.62–8.25	1.99	0.73–5.42
LDL	0.997	0.99–1.01	0.98	0.97–0.99	0.99	0.98–1.01	0.998	0.99–1.01
Initial glucose	1.01	1.00–1.01	1.004	0.998–1.01	1.01	1.00–1.02	1.01	1.00–1.02
SBP	1.01	0.996–1.02	1.004	0.99–1.02	0.99	0.97–1.01	0.98	0.96–0.996
SBP at f/u CT	0.99	0.97–1.02	1.02	0.99–1.04	1.04	1.00–1.08	1.04	1.01–1.07
HbA1c	0.72	0.49–1.05	1.27	0.88–1.83	0.62	0.35–1.08	0.71	0.42–1.22

*GA* glycated albumin, *END* early neurological deterioration, *mRS* modified Rankin Scale, *OR* odds ratio, *CI* confidence interval, *GCS* Glasgow coma scale, *NIHSS* National Institute of Health Stroke Scale, *LDL* low density lipoprotein, *SBP* systolic blood pressure, *f/u* follow-up, *CT* computed tomography, *HbA1c* glycated hemoglobin.

**Table 3:** Multivariate analysis showing impact of raw GA, GA/HbA1c and HbA1c on outcomes.

	Hematoma expansion		END		1-month mortality		3-month mRS 4–6	
	OR	95% CI	OR	95% CI	OR	95% CI	OR	95% CI
Raw GA	1.42	1.28–1.58	1.16	1.07–1.26	1.25	1.10–1.44	1.20	1.05–1.37
GA/HbA1c	11.13	5.49–22.55	2.57	1.53–4.30	4.95	1.95–12.54	3.06	1.36–6.89
HbA1c ≥ 6.5	3.05	1.41–6.62	2.90	1.33–6.33	0.70	0.19–2.55	2.35	0.80–6.89

*GA* glycated albumin, *HbA1c* glycated hemoglobin, *END* early neurological deterioration, *mRS* modified Rankin Scale, *OR* odds ratio, *CI* confidence interval.

**Figure 3:**
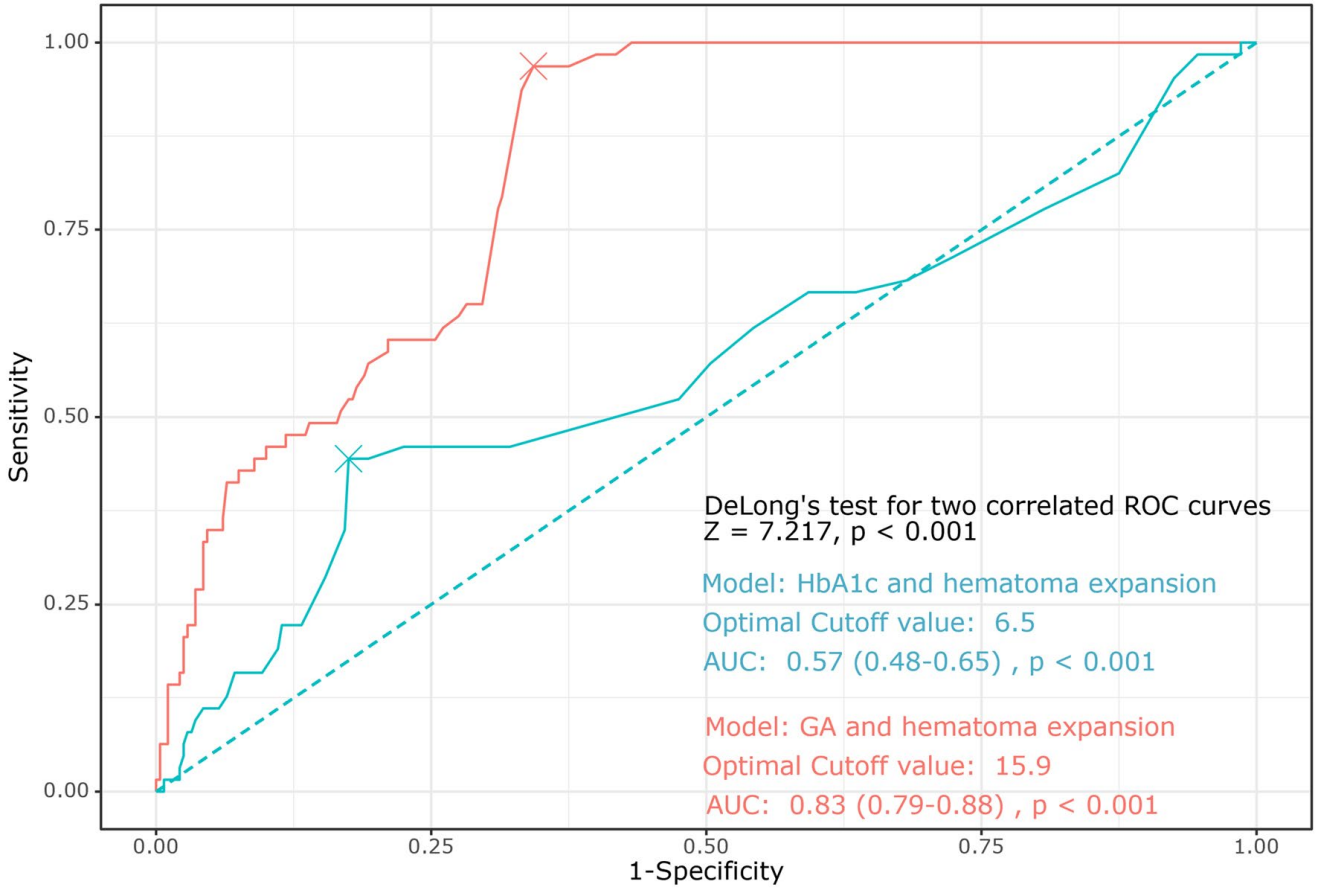
ROC curve showing the predictability and cutoff value of GA for hematoma expansion.

The ROC curve showed that the predictive ability of the GA level for hematoma expansion was good (AUC of GA: 0.83, 95% CI [0.79–0.88], p < 0.001]. However, the predictive ability of the HbA1c level for hematoma expansion was not better than that of the GA level (AUC of HbA1c 0.57, 95% CI [0.48–0.65], p for DeLong’s pairwise ROC comparison < 0.001). The cutoff values of GA and HbA1c levels were 15.9% and 6.5%, respectively, for hematoma expansion (Fig. 3).

## Discussion

The main findings of this study were as follows; (1) The higher GA group had more occurrence of hematoma expansion and poor short-term (END and 1-month mortality) and long-term outcomes (3-month mRS scores of 4–6); (2) Higher GA could be a strong predictor of hematoma expansion and other poor stroke outcomes in patients with ICH; (3) The predictive ability of GA for hematoma expansion was reliable, and the cutoff value of GA was 15.9%.

Oxidative stress may play a key role in brain damage after ICH^20^. Oxidative stress induces inflammatory reactions and produces free radicals after ICH^21,22^. During the inflammatory reaction after ICH, neutrophils and several proinflammatory cytokines generate reactive oxygen species and initiate the upregulation of metalloproteinase-9 expression. These pathomechanisms may alter cell integrity and increase cell membrane permeability, thereby leading to perihematomal edema, hematoma expansion via blood–brain barrier breakdown, vasospasm, and endothelial injury^23–26^. Several *in-vivo* and human studies have shown that exposure of cell cultures to rapid glycemic fluctuation produces more oxidative stress and cell damage than continuous hyperglycemia^8,9,27,28^. The present study may support the abovementioned notions and mechanisms concerning glycemic variability and ICH. Moreover, a high GA level could be a strong predictor of END, 1-month mortality, and 3-month mRS scores of 4–6 in this study. The sensitivity analysis that showed the relationship between the raw GA level and GA/HbA1c ratio and several outcomes supported our main results. These phenomena could also be explained by the aforementioned pathomechanisms. Several previous studies on ICH outcomes have focused on pre-stroke chronic hyperglycemia using HbA1c^5,29^, but the impact of pre-stroke glycemic variability on outcomes after ICH has not been investigated. We carefully suggested that our results could introduce the new marker for predicting prognosis, but also raise interest in the clinical implications of the effects of pre-stroke glycemic variability on outcomes after spontaneous ICH.

Therefore, our study revealed the predictive ability and cutoff value of GA for hematoma expansion in the acute phase of ICH. Compared with HbA1c, GA is a useful marker for monitoring glycemic variability, as GA more accurately reflects short-term glycemic fluctuation an postprandial, and fasting hyperglycemia^12,13,30^. Moreover, GA is not affected by several medical conditions, such as hematologic and renal diseases, which affect HbA1c and account for a large proportion of patients with ICH^31,32^. Notably, consistent with previous results^17^, we observed that more than half of patients with a higher GA level (47.1%) were observed among patients with well-controlled DM. Although clinical information of DM treatments prior to ICH were not available in this study, this finding indicated that glycemic variability could occur frequently even in patients with DM who had well-controlled glycemia and were without hyperglycemic symptoms. Given the evidence showing the reliability of pre-ICH glycemic control and the ease of calculation by blood sampling, we carefully considered that GA could be a reasonable parameter for predicting hematoma expansion and ICH outcomes in real-world practice of acute settings.

Interestingly, the HbA1c level, reflecting chronic hyperglycemia prior to ICH, was associated with hematoma expansion and END but not with delayed clinical outcomes in this study. Previous evidence showing the relationship between HbA1c and several prognostic outcomes of patients with ICH has been mixed. Some studies showed that HbA1c could be associated with worse long-term outcomes in patients with ICH^5,33^. However, a study with a large population (n = 21,116) showed an inverse association between HbA1c levels and short-term mortality, and a multicenter database study also showed negative associations among HbA1c, hematoma volume, and clinical outcomes^29,34^. These findings could explain that the effect of pre-stroke glycemic control on clinical outcomes may vary according to different phases of ICH onset. Additionally, initial random glucose levels, representing post-stroke glycemic status, was not associated with early outcomes but had a significant effect on long-term outcomes in this study. Post-stroke hyperglycemia also produced contradictory results, showing the association with the prognosis of stroke according to the ICH phase^4,33,35–37^. Taken together, HbA1c and post-stroke hyperglycemia have displayed various effects on prognosis according to stages of ICH, which is consistent with our findings. Although few researchers have investigated the impact of pre-stroke glycemic variability on the prognosis of ICH, our results carefully support the feasibility of using GA in clinical settings to predict short- and long-term outcomes following ICH. More studies are warranted to validate this finding.

Although the impact of pre-stroke glycemic variability using GA on prognosis after ICH was a novel finding, our study had several limitations. First, this study had a retrospective nature and a relatively small sample size, although we collected data consecutively. However, the strength of our study was including a single-center registry database, in which we routinely collected data on GA levels with a small missing rate for the study period and strictly performed BP control with a consistent institutional protocol. Second, although we controlled for several confounders in the statistical model, we could not completely control unmeasured confounding variables. Third, we did not consider medical conditions that increase protein metabolism and affect GA levels^14^. Particularly, information on alcohol consumption was not available in our database. Alcohol consumption is known to reduce the level of GA via altering glucose tolerance^38^ and has been identified as a potential risk factor for ICH^39^. Hence, caution should be observed in generalizing our results. Forth, markers for inflammation and oxidative stress which support patho-mechanism between glycemic variability and ICH outcomes were not available in our registry. Fortunately, high sensitive C-reactive protein (hs-CRP) could be collected in our study. The hs-CRP could identify low but persistent levels of inflammation^40^. In this study, we collected hs-CRP instead of interleukin and showed the indifference of the hs-CRP level between the higher GA and lower GA group. Further experimental study, such as *in-vivo* study should be warranted to demonstrate this issue.

In conclusion, this study suggests that pre-stroke glycemic variability is associated with hematoma expansion, END, 1-month mortality, and 3-month poor functional outcomes in spontaneous patients with ICH. Although further studies with a large population are warranted to address this issue, monitoring pre-stroke glycemic variability using GA after hospitalization, together with HbA1c, has reliability and feasibility to predict outcomes in patients presenting with ICH.

## Methods

### Population

We consecutively registered all acute intracranial hemorrhage patients within 24 h of onset between March 2016 and March 2022 at our institution. All enrolled patients underwent brain non-contrast computed tomography (NCCT) immediately after hospitalization in the emergency department. For the purpose of this study, we excluded the following patients: (1) patients with unavailable GA data; (2) patients without brain NCCT during hospitalization; (3) patients with secondary ICH (such as traumatic ICH, Moyamoya syndrome, aneurysmal rupture, arteriovenous malformations, tumor bleeding, hemorrhagic transformation after ischemic stroke); (4) patients with primary intraventricular hemorrhage (IVH); (4) pre-stroke modified Rankin scale (mRS) score > 1. We controlled blood pressure of enrolled patients in intensive care unit, a stroke predictor for hematoma expansion, according to American Heart Association guideline recommendations^41,42^.

### Ethic declaration

This study was conducted in accordance with the Helsiki declaration. The collection of clinical information with informed consent from the registry for monitoring and improving the quality and outcomes of stroke care was approved by the Institutional Review Board (IRB) of Chuncheon Sacred Heart Hospital (IRB no. 2013-03). The use of the registry database and the additional medical records for this study was approved by the IRB of Chuncheon Sacred Heart Hospital and provided the waiver for the need for informed consent from patients because of the study participants’ anonymity and minimal risk to patients (IRB no. 2020-03-010).

### Data collection and definition of parameters

We directly obtained demographic, clinical, laboratory, and outcome data from the registry database. The primary outcome measure was hematoma expansion defined as an absolute growth greater than 6 mL or a relative growth of more than 33% in the follow-up NCCT compared to the initial NCCT^43^. According to the institutional protocol, we performed routine follow-up CT 24 h and 7 days after ICH onset or at detecting neurological deterioration. We calculated ICH volume using the ABC/2 method, which is a widely used method^44^. An expert neurologist and neurosurgeon (S–H Lee and H K Choi) assessed all ICH volumes (interclass correlation coefficient = 0.88). The secondary outcome measures were early neurological deterioration (END), defined as a decrease in the Glasgow coma scale (GCS) score of > 3 points within 48 h of ICH onset^45^, and functional outcomes, defined as 3-month mRS scores. Poor functional outcome was defined as mRS score > 3, and good functional outcome was defined as mRS score ≤ 3^46^. We also collected several laboratoy test during hospitalization to registry database. In addition to the variables mentioned, we also collected hs-CRP as a marker for inflammation. The rationale for including hs-CRP was to investigate its potential association with glycemic variability and outcomes in patients with intracerebral hemorrhage.

### Measurement of GA level

Venous blood was collected from fasting patients within 8 h of hospitalization. Serum samples were collected and measured using an enzyme method with albumin-specific proteinase and ketoamine oxidase (Lucica GA-L; ASAHI KASEI PHARMA, Japan)^47^. Recent previous studies established that GA level ≥ 16.0% indicates the presence of glycemic variability prior to ICH based on the following equation of HbA1c and GA: HbA1c = 0.216 × GA + 2.978^16–19,48^. According to this conversion equation, substituting HbA1c of 6.5% yields GA of approximately 16.0%. The previous studies indicated the cutoff value of GA to identify DM. A study in Taiwan (n = 2192) described a cutoff point of GA ≥ 14.5% for DM. In addition, considering the value of 6.5% of HbA1c, the corresponding GA was 16.5%. Other study in Japan (n = 1575) also described the cutoff point of GA ≥ 15.5%. Therefore, we established that a GA level ≥ 16.0% reflected the presence of glycemic variability prior to ischemic stroke based on the abovementioned equation of HbA1c and GA. Given the cutoff point presented in previous studies and based on the above equation, we divided the population into lower (GA < 16.0%) and higher GA (GA ≥ 16.0%) groups. The equation, HbA1c = 0.216 × GA + 2.978, provide a mathematical relationship between these two measures. We considered the cutoff value of 16.0% to reflect the presence of glycemic variability prior to stroke based on this conversion equation. It is important to note that chosen cutoff value aligns with values described in previous studies investigating GA in different population. By using this cutoff value, we aimed to indemnity patients with higher pre-stroke glycemic variability. Moreover, while the equation derived from the HbA1c-GA correlation does not directly explain the differences in sensitivity and specificity, it highlights the relationship between these two measures.

### Statistical analysis

Statistical analyses were performed using IBM SPSS version 21.0 software (IBM Corporation, Armonk, NY, USA) and R version 4.0.3 (R core Team 2020; R Foundation for Statistical Computing, Vienna, Austria). Summary statistics are presented as the number of patients (percentage) for categorical variables and as the mean ± standard deviation (SD) or median [interquartile range (IQR)] for continuous variables. Group comparisons were made using the Pearson’s chi-squared test for categorical variables and the Student’s *t* test or Mann–Whitney *U* test for continuous variables, as appropriate.

Regarding the primary and secondary outcome measures, the higher GA group and lower GA group were compared using the Pearson’s chi-squared test for categorical variables and the Student’s *t* test or Mann–Whitney *U* test for continuous variables. In our study, we conducted a multivariable analysis to assess the association between glycated albumin (GA) and hematoma expansion, early neurological deterioration (END), and functional outcomes, while controlling for potential confounders. During the variable selection process, we initially included a wide range of covariates in the multivariable models. However, to prevent overfitting and ensure robustness of the analysis, we applied a conservative approach and excluded variables that did not meet a specific statistical threshold (e.g. p-value < 0.1) in the univariate analysis. Variables that did not reach this threshold were deemed less likely to have a significant independent association with the outcomes of interest. By excluding these variables, we aimed to focus on the most relevant factors associated with our study outcomes. The selected covariates were chosen based on their clinical relevance, statistical significance in the univariate analysis, and consideration of prior literature. our analysis included important confounders that are commonly recognized in the field of intracerebral hemorrhage and glycemic control. Crude and adjusted odds ratios (ORs) and 95% confidence intervals (CIs) were estimated.

To assess the predictive ability of GA and HbA1c levels for hematoma expansion, we constructed a receiver operating characteristic (ROC) curve using the ‘pROC’ package of R. The 95% CI for the area under the curve (AUC) and p value were calculated using the Delong’s test. The cutoff values of GA and HbA1c levels for hematoma expansion were calculated using the Youden index.

In a sensitivity analysis, we also analyzed the impact of the GA/HbA1c ratio and the raw GA level on outcomes using a logistic regression model.

### Supplementary Information

Supplementary Tables.

## Data Availability

All datasets generated and/or analyzed during the current study are not publicly available as use of the data requires ethical approval. To inquire access to the study data, contact the corresponding author.
