# Supplementary material for: Pre-stroke glycemic variability estimated by glycated albumin predicts hematoma expansion and poor outcomes in patients with spontaneous intracerebral hemorrhage
Source: Sci Rep. 2023 Aug 8;13:12848. doi: 10.1038/s41598-023-40109-5 (PMC10409739; doi:10.1038/s41598-023-40109-5)
Supplement: Supplementary file 1 — Supplementary Tables. [file 41598_2023_40109_MOESM1_ESM.docx]

Supplementary table 1. Multivariate analysis showing impact of raw GA level on outcomes

|  | Hematoma expansion | | END | | 1-month mortality | | 3-month mRS 4-6 | |
| --- | --- | --- | --- | --- | --- | --- | --- | --- |
|  | OR | 95% CI | OR | 95% CI | OR | 95% CI | OR | 95% CI |
| Raw GA | 1.42 | 1.28-1.58 | 1.16 | 1.07-1.26 | 1.25 | 1.10-1.44 | 1.20 | 1.05-1.37 |
| Age | 0.98 | 0.96-1.01 | 1.01 | 0.98-1.04 | 1.06 | 1.02-1.11 | 1.04 | 1.01-1.08 |
| Male | 0.79 | 0.38-1.66 | 0.67 | 0.32-1.40 | 2.19 | 0.66-7.23 | 1.52 | 0.64-3.60 |
| Initial GCS | 0.86 | 0.62-1.20 | 0.83 | 0.60-1.14 | 0.51 | 0.31-0.83 | 0.50 | 0.33-0.77 |
| Initial NIHSS | 0.92 | 0.73-1.16 | 0.86 | 0.69-1.07 | 1.01 | 0.78-1.29 | 1.004 | 0.73-1.38 |
| prior stroke | 0.55 | 0.21-1.44 | 0.69 | 0.28-.1.71 | 0.79 | 0.22-2.83 | 1.50 | 0.49-4.57 |
| Hypertension | 2.29 | 1.06-4.95 | 2.06 | 0.96-4.42 | 0.86 | 0.27-2.74 | 0.47 | 0.19-1.13 |
| Diabetes mellitus | 0.66 | 0.25-1.71 | 1.44 | 0.65-3.21 | 0.91 | 0.23-3.65 | 3.17 | 1.09-9.16 |
| Prior antithrombotics | 1.65 | 0.69-3.93 | 0.54 | 0.24-1.25 | 2.38 | 0.65-8.70 | 1.71 | 0.61-4.76 |
| LDL | 0.999 | 0.99-1.01 | 0.98 | 0.97-0.99 | 0.99 | 0.98-1.01 | 0.999 | 0.99-1.01 |
| Initial glucose | 1.001 | 0.99-1.01 | 1.002 | 0.996-1.01 | 1.01 | 1.00-1.02 | 1.01 | 0.996-1.01 |
| SBP | 1.02 | 1.00-1.03 | 1.01 | 0.99-1.02 | 0.998 | 0.98-1.02 | 0.98 | 0.96-0.998 |
| SBP at f/u CT | 0.98 | 0.96-1.01 | 1.01 | 0.99-1.04 | 1.03 | 0.999-1.06 | 1.04 | 1.00-1.07 |
| HbA1c | 0.37 | 0.23-0.62 | 1.07 | 0.71-1.60 | 0.46 | 0.25-0.84 | 0.64 | 0.35-1.15 |

Supplementary table 2. Multivariate analysis showing impact of GA/HbA1c ratio on outcomes

|  | Hematoma expansion | | END | | 1-month mortality | | 3-month mRS 4-6 | |
| --- | --- | --- | --- | --- | --- | --- | --- | --- |
|  | OR | 95% CI | OR | 95% CI | OR | 95% CI | OR | 95% CI |
| GA/HbA1c | 11.13 | 5.49-22.55 | 2.57 | 1.53-4.30 | 4.95 | 1.95-12.54 | 3.06 | 1.36-6.89 |
| Age | 0.98 | 0.96-1.01 | 1.01 | 0.99-1.04 | 1.06 | 1.02-1.10 | 1.04 | 1.01-1.08 |
| Male | 0.79 | 0.37-1.67 | 0.76 | 0.37-1.54 | 2.24 | 0.67-7.46 | 1.54 | 0.65-3.63 |
| Initial GCS | 0.83 | 0.59-1.16 | 0.80 | 0.58-1.09 | 0.48 | 0.29-0.80 | 0.49 | 0.32-0.76 |
| Initial NIHSS | 0.89 | 0.70-1.13 | 0.82 | 0.66-1.03 | 0.99 | 0.77-1.27 | 0.995 | 0.72-1.37 |
| prior stroke | 0.58 | 0.22-1.52 | 0.80 | 0.33-1.93 | 0.76 | 0.22-2.63 | 1.47 | 0.48-4.47 |
| Hypertension | 2.32 | 1.07-5.04 | 1.79 | 0.86-3.70 | 0.87 | 0.28-2.75 | 0.47 | 0.19-1.12 |
| Diabetes mellitus | 0.71 | 0.28-1.78 | 1.83 | 0.84-3.95 | 0.84 | 0.21-3.39 | 3.27 | 1.18-9.07 |
| Prior antithrombotics | 1.69 | 0.71-4.06 | 0.59 | 0.26-1.33 | 2.52 | 0.68-9.32 | 1.77 | 0.63-4.94 |
| LDL | 1.00 | 0.99-1.01 | 0.98 | 0.97-0.995 | 0.99 | 0.98-1.01 | 0.999 | 0.99-1.01 |
| Initial glucose | 1.002 | 0.997-1.01 | 1.01 | 1.002-1.01 | 1.01 | 0.999-1.01 | 1.01 | 0.999-1.01 |
| SBP | 1.01 | 1.00-1.03 | 1.01 | 0.995-1.02 | 0.996 | 0.98-1.02 | 0.98 | 0.96-0.997 |
| SBP at f/u CT | 0.98 | 0.96-1.01 | 1.01 | 0.99-1.04 | 1.04 | 1.00-1.07 | 1.04 | 1.00-1.07 |

Supplementary table 3. Multivariate analysis showing impact of HbA1c on outcomes

|  | Hematoma expansion | | END | | 1-month mortality | | 3-month mRS 4-6 | |
| --- | --- | --- | --- | --- | --- | --- | --- | --- |
|  | OR | 95% CI | OR | 95% CI | OR | 95% CI | OR | 95% CI |
| HbA1c≥6.5 | 3.05 | 1.41-6.62 | 2.90 | 1.33-6.33 | 0.70 | 0.19-2.55 | 2.35 | 0.80-6.89 |
| Age | 0.995 | 0.97-1.02 | 1.01 | 0.99-1.04 | 1.06 | 1.02-1.10 | 1.05 | 1.01-1.08 |
| Male | 0.82 | 0.43-1.59 | 0.73 | 0.37-1.47 | 1.86 | 0.61-5.69 | 1.60 | 0.69-3.71 |
| Initial GCS | 0.87 | 0.66-1.15 | 0.83 | 0.61-1.12 | 0.57 | 0.36-0.90 | 0.51 | 0.33-0.78 |
| Initial NIHSS | 0.94 | 0.78-1.13 | 0.86 | 0.70-1.06 | 1.02 | 0.80-1.29 | 1.01 | 0.74-1.39 |
| prior stroke | 0.63 | 0.27-1.47 | 0.82 | 0.34-1.95 | 0.76 | 0.23-2.47 | 1.58 | 0.51-4.91 |
| Hypertension | 1.88 | 0.95-3.73 | 1.77 | 0.86-3.68 | 0.85 | 0.29-2.52 | 0.45 | 0.19-1.08 |
| Diabetes mellitus | 0.89 | 0.40-2.01 | 1.61 | 0.73-3.56 | 1.65 | 0.47-5.80 | 2.84 | 0.96-8.38 |
| Prior antithrombotics | 1.17 | 0.55-2.49 | 0.56 | 0.25-1.25 | 1.32 | 0.40-4.35 | 1.84 | 0.68-4.95 |
| LDL | 0.997 | 0.99-1.01 | 0.98 | 0.97-0.99 | 0.99 | 0.97-1.01 | 0.998 | 0.99-1.01 |
| Initial glucose | 1.003 | 0.998-1.01 | 1.01 | 1.00-1.01 | 1.01 | 1.00-1.02 | 1.01 | 0.999-1.01 |
| SBP | 1.01 | 0.995-1.02 | 1.01 | 0.99-1.02 | 0.998 | 0.98-1.02 | 0.98 | 0.96-0.996 |
| SBP at f/u CT | 0.99 | 0.97-1.02 | 1.01 | 0.99-1.04 | 1.03 | 0.998-1.07 | 1.04 | 1.00-1.07 |
